# Daily Prey Consumption and Functional Response of *Orius insidiosus*: Implications for Biological Control of *Scirtothrips dorsalis* in Strawberries

**DOI:** 10.3390/insects16020205

**Published:** 2025-02-13

**Authors:** Lovely Adhikary, Hugh Adam Smith, Sriyanka Lahiri

**Affiliations:** Department of Entomology and Nematology, Gulf Coast Research and Education Center, University of Florida, Wimauma, FL 33598, USA; hughasmith@ufl.edu (H.A.S.); lahiris@ufl.edu (S.L.)

**Keywords:** chilli thrips, prey density, predator behavior, predator–prey interaction

## Abstract

Strawberry is an important specialty crop grown in Florida. Recently, chilli thrips, *Scirtothrips dorsalis* Hood (Thysanoptera: Thripidae), have become a significant threat to Florida strawberry production. Pesticide applications are not always recommended because of the development of insecticidal resistance. Biological control can be a viable control option for this pest. However, the management of *S. dorsalis* using predatory bug *Orius insidiosus* Say (Hemiptera: Anthocoridae) has never been explored in strawberries. Therefore, this study’s aim was to evaluate the predation efficacy of *O. insidiosus* through daily consumption rate and the functional response while preying on *S. dorsalis*. The results suggest that adult *O. insidiosus* has a significantly higher daily consumption rate than fifth-instar nymphs when feeding on both life stages of *S. dorsalis*. A Type II functional response was expressed by *O. insidiosus* when feeding on larval and adult *S. dorsalis,* indicating that the predation rate can increase with the prey density before it reaches a saturation point. The attack rates (a) and prey handling time (T_h_) were also computed on second-instar larvae and adult *S. dorsalis*, respectively. The results indicate that *O. insidiosus* can be used as an augmentation biocontrol agent for *S. dorsalis* management in strawberries.

## Simple Summary

Chilli thrips, *Scirtothrips dorsalis* Hood, have recently become a major pest of Florida strawberries after its invasion in the US. Biological control is an important measure in the integrated management of this pest. However, biological control of *S. dorsalis* heavily depends on the release of predatory mites. The predatory insect *Orius insidiosus* can also be a potential biocontrol agent for *S. dorsalis*. This study observed the daily prey consumption rate and type of functional response expressed by *O. insidiosus* with increased prey density. Results showed that *O. insidiosus* adults consumed more prey than fifth-instar nymphs and showed Type II functional response preying on both adult and immature life stages of *S. dorsalis*. The results revealed that *O. insidiosus* has the potential to suppress the population of *S. dorsalis*.

## 1. Introduction

Strawberry *Fragaria × ananassa* Duchesne (Rosales: Rosaceae) is an important horticultural crop in Florida, U.S.A., cultivated on over 5200 hectares and generating a revenue of about USD 511 million annually [1]. Various arthropod pests, including tarnished plant bug *Lygus lineolaris* (Pallisot de Beauvois) (Hemiptera: Miridae), two-spotted spider mite *Tetranychus urticae* (Koch) (Acari: Tetranychidae), and cyclamen mite *Phytonemus pallidus* (Banks) (Acari: Tarsonemidae), infest open-field strawberries throughout the growing season in Florida [2]. Lately, the invasive chilli thrips, *Scirtothrips dorsalis* Hood (Thysanoptera: Thripidae), has become a significant pest of Florida strawberries after its introduction from Southeast Asia in 2005 [3]. *Scirtothrips dorsalis* infestation on strawberry plants produces dark malformed leaves and bronzed, cracked fruits. The management of *S. dorsalis* is mainly dependent on insecticide applications; however, excessive insecticide use can result in resistant *S. dorsalis* populations [4] and harm the non-target organisms [5]. Biological control may provide an alternative or complementary strategy to insecticide application for managing *S. dorsalis.* Biological control can be defined as the use of living organisms to reduce the pest population, making it less prevalent than it would be naturally [6].

*Orius insidiosus* Say (Hemiptera: Anthocoridae), commonly known as the minute pirate bug, is a generalist predator [7] abundant in many parts of the United States that feeds on many important insect pests. *Orius* spp. are commercially available and can be used in augmentation biocontrol in greenhouses or open-field agriculture to manage different thrips species [8]. For example, a high density *Orius* spp. was found to control the population of *Thrips palmi* Karny (Thysanoptera: Thripidae) in greenhouse eggplants in Japan [9]. The predation efficacy of *Orius* spp. was observed against *Frankliniella occidentalis* Pergande (Thysanoptera: Thripidae) and *Thrips tabaci* Lindeman (Thysanoptera: Thripidae) on sweet pepper in the greenhouse [8]. *Orius insidious* was also an effective predator of *F. occidentalis* on chilli pepper in the field [10].

*Orius laevigatus* (Fieber) (Hemiptera: Anthocoridae), native to Europe, was efficient in controlling *F. occidentalis* in strawberries [11] and in that study, the number of *F. occidentalis* per strawberry flower reduced when the predators were released compared to the control cages without the predators. However, very few studies have evaluated *Orius* spp. for managing *S. dorsalis*. *Orius laevigatus* and *Amblyseius swirskii* Athias-Henriot (Arachnida: Phytoseiidae) effectively controlled *S. dorsalis* on chilli pepper in Sri Lanka [12]. Another study found that *O. insidious* in association with *A. swirskii* effectively reduced *S. dorsalis* population and *O*. *insidious* was more efficient in controlling adult thrips than *A. swirskii* [13].

Many reports have shown the efficiency of anthocorid bugs feeding on soft-bodied insects, including thrips [14]. However, pest control by predators depends on many factors, including the predator’s biological and behavioral traits [15]. One important method to evaluate a biological control agent’s efficacy is to ascertain its response to the changes in prey densities or understand its functional response [15].

The functional response of a predator can be defined as a relationship between prey density and prey consumption [16]. It is commonly used as a primary indicator of the ability of a candidate biocontrol agent to suppress the pest population below the threshold level [17,18]. Three types of functional responses were defined by Holling 1959 [19]. In Type I, predators demonstrate a linear increase in the consumption rate with an increase in prey density; in Type II, predators gradually decrease the prey consumption as the prey density increases; and in Type III, predators demonstrate a rise in consumption rate which follows a sigmoidal response shape. Functional response helps one to understand two crucial parameters of predators, i.e., prey handling time and searching ability [20].

Previous studies using *Orius laevigatus* and *Orius majusculus* (Reuter) (Hemiptera: Anthocoridae) showed a Type II functional response when preying on two aphid species, *Aphid gossypii* Glover and *Macrosiphum euphorbiae* Thomas (Hemiptera: Aphididae) [21]. In another study, *O. laevigatus* and *O. majusculus* were tested for functional responses on whitefly *Bemisia tabaci* (Gennadius) (Hemiptera: Aleyrodidae) pupae (fourth-instar nymphs) and *F. occidentalis* second-instar larvae in greenhouse vegetables. This study also found a Type II functional response by both *Orius* spp. [22]. A previous study with *Orius sauteri* (Hemiptera: Anthocoridae) (Poppius) showed a Type II functional response towards *Thrips palmi* [23].

In Florida strawberries, the main biological control agents used for the management of *S. dorsalis* and *T. urticae* are predatory mites. The potential of *Orius* spp., which are known thrips predators, to control *S. dorsalis* in strawberries has not been studied before. Therefore, the objective of the study was to (1) determine the rate of daily prey consumption by adult and fifth-instar nymphs of predatory insect *Orius insidiosus* and (2) determine the type of functional response expressed by *O. insidiosus* with increased prey density. As both larvae and adults of *S. dorsalis* are damaging life stages to strawberry plants, both life stages were tested in this experiment. On the other hand, all life stages of *O. insidiosus* are predatory, which is why both the fifth-instar nymph and adults were tested for their daily prey consumption rate.

## 2. Materials and Methods

*Laboratory colony of S. dorsalis*: Upland cotton plants, *Gossypium hirsutum* L. (Malvaceae), were grown from seed in pots (10.2 cm × 8.9 cm, Greenhouse Megastore, Danville, IL, USA) with soil (BWI Pro-Mix 2.8 cf; BWI Companies, Nash, TX, USA) in a growth room at the Gulf Coast Research and Education Center (GCREC) (27.7604, −82.2275), for maintaining an *S. dorsalis* colony. The cotton plants infested with *S. dorsalis* were kept inside cages (Bug Dorm, BioQuip Products Inc. Compton, CA, USA) at 25 ± 5 °C and 60 ± 5% RH in a 16L:8D hour photophase. New cotton plants were introduced inside the previously infested cages every two weeks. All plants were watered as needed every alternate day and fertilized (J R Peter’s Jacks General All-Purpose Fertilizer, Allentown, PA, USA) once in two weeks.

*Strawberry plant husbandry*: Bare-root transplants of the strawberry cultivar “Florida Brilliance” (US Patent PP 30,564) [24] were obtained from a commercial nursery (Crown Nursery LLC, Red Bluff, CA, USA). The transplants were planted in pots of the same size as described in the previous section and maintained accordingly. Plants were kept inside a greenhouse at GCREC with a temperature of 25 ± 5 °C and 75 ± 5% relative humidity (HOBO U23 Prov2; Onset Computer Corporation, Bourne, MA, USA) and a natural photoperiod. After almost three weeks, when the plants had at least five full-grown trifoliates, the leaves were used in the experiment.

*Rate of daily prey consumption*: All bioassays were conducted in the Strawberry and Small Fruit Entomology Lab at Gulf Coast Research and Education Center (27.712490°, −82.302322°). Each experimental unit was a polypropylene Petri dish with a 5 cm diameter (Fisherbrand, Suwanee, GA, USA). *Orius insidiosus* were purchased from commercial sources (BioBee Ltd., Salisbury, MD, USA and Crop Defenders Ltd., Plymouth, MI, USA). All the predators used in the study were starved for 24 h upon arrival before the experiment and provided with water only. One young strawberry leaf was fitted inside the Petri dish. A small piece of moist filter paper was provided as a moisture source (5.5 cm diameter Whatman qualitative filter paper, Sigma-Aldrich^®^, Saint Louis, MO, USA). Ten adult *S. dorsalis* were collected from the existing colony by aspiration and released in the Petri dish. One individual *O. insidiosus* adult was released as a predator in the Petri dish. A similar experimental arena was prepared for the fifth-instar nymph of *O. insidiosus,* with ten *S. dorsalis* provided as prey. Two life stages, i.e., second-instar larvae and adults of *S. dorsalis*, were tested separately in these arenas as prey for *O. insidiosus*. After releasing both prey and predator, the Petri dishes were sealed with Parafilm™ (Fisher Scientific, Hampton, NH, USA) and labeled with the date. The Petri dishes were maintained at 25 °C and 65% RH in a 16L: 8D photoperiod. Petri dishes were examined under a stereomicroscope (Stemi 508, Carl Zeiss, Germany) at 40× magnification after 24 h and the number of prey consumed was recorded. Each experiment was repeated three times with ten replications per treatment.

*Functional Response*: The experimental arena was a 5 cm polypropylene Petri dish (Fisherband, Suwanee, GA, USA). Each Petri dish had a strawberry leaf (Florida Brilliance) and a moist filter paper (5.5 cm diameter Whatman qualitative filter paper, Sigma-Aldrich^®^, MO, USA) as a moisture source. Prey densities of 5, 10, and 20 *S. dorsalis* adult and second-instar larvae were used in the study. Adult *Orius insidiosus* were starved for 24 h before the experiment and provided with water only. One adult predator was released into the arena with the prey. After 24 h, the prey consumption was evaluated under a stereomicroscope (Stemi 508, Carl Zeiss, Germany) at 40× magnification and the mortality data were recorded. The experiment was performed at 25 ± 1 °C and 65 ± 5% RH in a 16L: 8D photoperiod. The experiment was conducted with ten replicates for each prey density and repeated three times.

*Data analysis*: The difference between prey consumption by adults and 5th-instar nymphs of *O. insidiosus* was determined using a generalized linear mixed model (Proc Glimmix) in SAS OnDemand for Academics web platform (SAS Ins. Cary, NC, USA). Mean separation was performed using Tukey’s HSD at α = 0.05.

Functional response was determined by the number of prey consumed at different prey densities. Data analysis was performed in two steps. In the first step, logistic regression analysis was performed to determine the type of functional response expressed by *O*. *insidiosus*. The linear coefficient was determined by logistic regression of the proportion of the consumed (N_a_/N_0_) prey as a function of prey offered [25].

(1) $$\frac{N_{a}}{N_{0}}=\frac{\exp(P_{0}+P_{1}N_{0}^{1}+P_{2}N_{0}^{2}+P_{3}N_{0}^{3})}{1+\exp(P_{0}+P_{1}N_{0}^{1}+P_{2}N_{0}^{2}+P_{3}N_{0}^{3})}$$

where N_a_ = prey consumed, N_0_ = initial prey offered, P_0_, P_1_, P_2_, and P_3_ are the intercept, linear, quadratic, and cubic co-efficients, respectively, and N_a_/N_0_ is the probability of prey consumed. These parameters were calculated by Proc CATMOD in SAS OnDemand for Academics web platform (SAS Ins. Cary, NC, USA). According to Juliano, [26] when P_1_ < 0 and P_2_ > 0, the proportion of consumed prey decreased gradually as the initial number of prey increased. This phenomenon follows a Type II functional response. The maximum likelihood estimates show how the independent variable, which was the different initial prey counts, influences the likelihood of different predation outcomes. This study divided the initial prey count into three levels, 5 as low, 10 as medium, and 20 as high, and the outcome may represent different predator behaviors: low, medium, and high predation activity. In our study, the model considered the medium initial prey density (10 *S. dorsalis* per *O. insidiosus*) as the reference level.

In the second step of the analysis, after the type of functional relationship had been determined, the Holling disc equation (Equation (2)) was used to understand the parameters of predator attack rate or instantaneous searching rate (a) and handling time (h). A non-linear least square regression was performed by Proc NLIN in SAS OnDemand for Academics web platform to estimate these parameters.

(2) $$N_{a}=\frac{\mathrm{aT}N_{0}}{1+T_{h}N_{0}}$$

where N_a_ = number of prey consumed; N_0_ = initial number of prey; a = searching efficiency or attack rate; T = total time available for the predator exposure, which was 24 h in our experiment; and T_h_ = handling time per prey. The attack rate (a) and handling time (T_h_) were analyzed after the determination of Type II functional response following the Holling disc equation [19].

## 3. Results

### 3.1. Daily Consumption Rate of O. insidiosus on S. dorsalis

**Figure 1:**
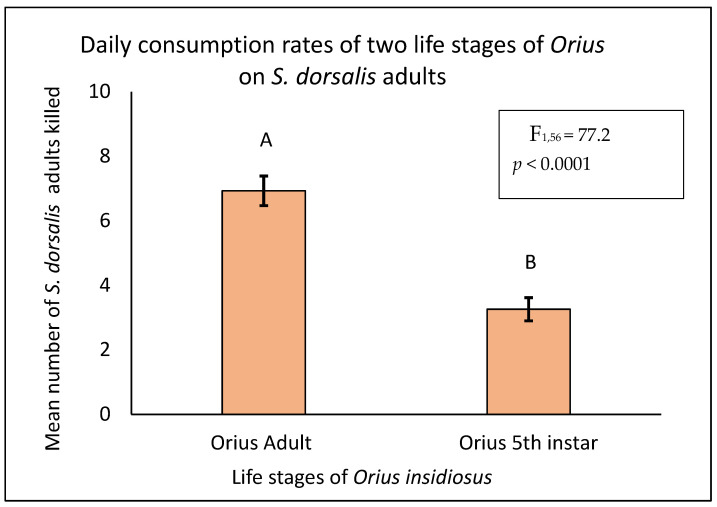
Mean number of *S. dorsalis* adults consumed in 24 h by adult and 5th-instar nymphs of *Orius insidiosus* (Proc Glimmix, α = 0.05, SAS Institute Inc., Cary, NC, USA). The different letters represent the significant difference in prey consumption.

**Figure 2:**
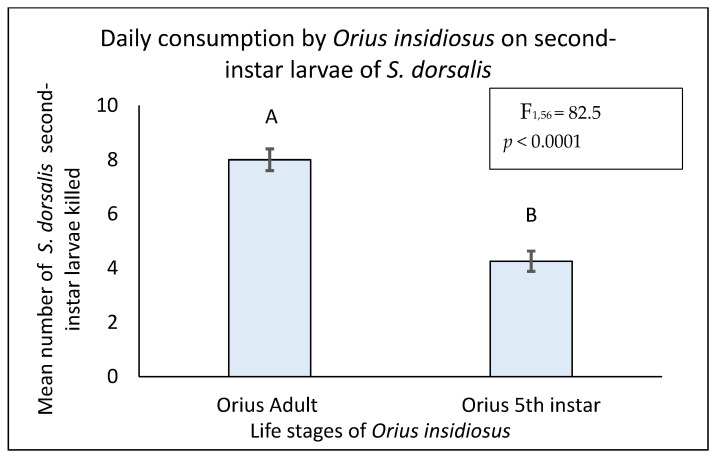
Mean number of second-instar larvae of *S. dorsalis* consumed in 24 h by adult and 5th-instar nymphs of *Orius insidiosus* (Proc Glimmixα = 0.05, SAS Institute Inc., Cary, NC, USA). The different letters represent the significant difference in prey consumption.

In both experimental settings, adult *O. insidiosus* consumed a significantly higher number of prey than fifth-instar nymphs. In the experimental arena where the adult *S. dorsalis* was offered as prey, the adult *O. insidiosus* consumed a significantly higher number of prey compared to the fifth-instar nymphs (F = 77.2, df = 1,56, *p* < 0.0001) (Figure 1). A similar result was obtained when the second-instar larvae of *S. dorsalis* were offered as prey (F = 82.5, df = 1,56, *p* < 0.0001) (Figure 2).

### 3.2. Functional Response of O. insidiosus on S. dorsalis (Second-Instar Larvae)

The intercept (P_0_) represents the baseline log odds when all other variables were at their reference level, which is the medium initial prey category in this case. When the second-instar larvae were offered as prey, the first possible outcome was −1.22003 (*p* < 0.0001) (Table 1), suggesting that the baseline likelihood of this outcome was low before considering changes in prey availability, whereas possible outcome 2 was 0.1951 (*p* = 0.033). This was positive and statistically significant above zero, indicating a higher baseline likelihood of this outcome relative to the reference category (Table 1). This result suggests that predators will be more likely to engage in predation activity when the initial prey is at the reference level (10 *S. dorsalis* per *O. insidiosus*).

The first possible outcome of the linear co-efficient (P_1_) showed that certain predation outcomes decreased as the estimate was negative and not significant (−0.2338 *p* = 0.1441). This result indicated that high initial prey density had very little effect on predator behavior (Table 1). In comparison, possible outcome 2, regarding the high initial prey density, showed an estimate of −0.2158 (*p* = 0.0412). This result indicated that the odds of the outcome were significantly low, relative to the reference category. This suggests that *O. insidiosus* might be less likely to consume a higher amount of prey when initial prey density is high.

The two possible outcomes of the quadratic co-efficient (P_2_) were 0.6607 (*p* = 0.0035) and 0.7211 (*p* = <0.001) These two estimates were positive and significant, which indicates the increase in the log odds of different biological outcomes such as partial or complete predation of available prey compared to the reference category. This suggests that when the initial prey *S. dorsalis* numbers were low, the predator was more likely to engage in predation.

### 3.3. Functional Response of O. insidiosus on S. dorsalis (Adults)

The possible outcomes of intercept (P_0_) were 0.8500 (*p* < 0.0001) and 0.3973 (*p* = 0.0195), respectively, which were positive and significantly above zero. These results indicate that *O. insidiosus* would show some predacious activity without considering the initial prey density when adult *S. dorsalis* is offered as prey (Table 2).

The linear co-efficient (P_1_) showed both possible outcomes as negative and statistically significant: −0.4989 (*p* = 0.004) and −2.3432 (*p* = <0.0001) (Table 2). These values indicated that the predator *O. insidiosus* was less likely to engage in predation activity when the initial prey density was high.

The quadratic co-efficient (P_2_) showed both of the possible outcomes as positive and significant, 0.6698 (*p* = 0.0205) and 1.9053, (*p* < 0.0001), respectively. These positive and significant estimates indicated an increase in the log odds of the predation activity, suggesting that when prey availability is low, the predator will likely acquire more prey.

In summary, the negative values in the high initial prey density in both cases of second-instar larvae and adult *S. dorsalis* indicate that *O. insidiosus* was less likely to exploit all of the prey. However, when the initial prey was low, the predator was more likely to maximize the predation rate. This suggests a Type II functional response where predation activity increases rapidly at a low prey density and decreases towards a higher initial prey density.

The attack rates of *O. insidiosus* on the second-instar larvae and adult *S. dorsalis* were 0.0342 ± 0.0018 h^−1^ and 0.0335 ± 0.0042 h^−1^ per unit of prey density, respectively. The prey handling time was 0.01 ± 0.00 h and 0.8135 ± 0.2419 h for second-instar larvae and adult thrips, respectively (Table 3).

## 4. Discussion

### 4.1. Daily Prey Consumption

*Orius insidiosus* adults demonstrated higher prey consumption than the fifth-instar nymphs when both adult and second-instar larvae of *S. dorsalis* were provided as prey. This result could be due to their better maneuverability than the *O. insidiosus* nymphal stages [26]. Predators often prefer to prey on insects with less mobility, which could explain the higher predation rate on second-instar larvae of *S. dorsalis*. Another study found that *Orius laevigatus* and *Orius majusculus* were feeding more on sedentary leaf-inhabiting thrips species *Echinothrips americanus* (Thripidae: Thysanoptera) than mobile thrips like *F. occidentalis* [27]. A similar result was found by Isenhour and Yeargan [28] where the adult *O. insidiosus* killed more soybean thrips, *Sericothrips variabilis* (Beach), compared to *O. insidiosus* nymphal instars. A lower predation rate by the juvenile stage of a predator could be due to their smaller body size, slower movement, and long prey handling time [29].

### 4.2. Functional Response of O. insidiosus 

The determination of the functional response of any biocontrol agent will advance our understanding of their potency in the regulation of pest population [30]. Previous studies have indicated that most arthropod predators exhibit a Type II functional response [31]. Similarly, our results indicated Type II functional response by *O*. *insidiosus* to both life stages of *S*. *dorsalis* (Table 1 and Table 2).

Other *Orius* spp. have previously shown Type II functional responses against various pests. For example, *Orius nijer* Wolff (Hemiptera: Anthocoridae) and *Orius minutus* L. (Hemiptera: Anthocoridae) have shown Type II functional responses when *Tetranychus urticae* adults and second-instar larvae of onion thrips, *Thrips tabaci* Lindeman, were offered as prey [18]. *Orius sauteri* (Poppius) also showed a Type II functional response when fed on *Megalurothrips usiatus* (Bagnall) (Thysanoptera: Thripidae) [32]. *Orius albidipennis* (Reuter) (Hemiptera: Anthocoridae) exhibited a Type II functional response when preying on *Megalurothrips sjostedji* Trybom [33]. Another important predator that showed a Type II functional response was the Asian ladybird beetle *Harmonia axyridis* (Pallas) (Coleoptera: Coccinellidae) when feeding on various species of aphids [34].

Predators showing low handling time and high attack rates are believed to be more efficient biological control agents [18]. However, in our study, the attack rate by *O. insidiosus* was low, observed as 0.0342 ± 0.0018 and 0.0335 ± 0.004 on second-instar larvae and adults of *S. dorsalis*, respectively (Table 3). As discussed before, the attack rate of the predator can be restricted by handling time [30]. Various thrips species often show anti-predatory behavior, including waging of the abdomen and secretion of anal fluids [35,36] which could have negatively impacted the handling time of the predator. However, in the field, predators can face limitations due to searching area and time [30,37,38]. Handling time refers to a predator’s time to overpower, process, and consume the prey [19]. Low handling implies that the predator can quickly process and consume the prey faster, ultimately increasing the feeding rate. In the case of Type II functional response, low handling time can lead to a higher predation rate [39] because the predator is less constrained with time, allowing it to consume more prey before reaching saturation. From the result of our study, we can say that *O. insidiosus* will consume prey relatively quickly when the prey density is low. However, when prey density increases, predation becomes limited because of handling time. The predators that show Type II functional response can be used in inundative biological control to reduce the pest population [40].

Functional response is a vital tool in understanding predators’ efficacy, but it cannot be the only measure of the success or failure of a biological control agent. Other biotic and abiotic factors such as host patchiness, intrinsic growth rates, predation and competition, other environmental factors and the ability to survive on alternate food sources, can also significantly affect the adeptness of a predator in managing a pest population [41,42]. Functional response studies performed in laboratory conditions may lack the ability to determine predation characteristics in field conditions [43]. Previous studies have found differences in functional response in the predatory stink bug *Podisus maculiventris* (Say) (Hemiptera: Pentatomidae) in laboratory and field conditions [44]. Some reasons for this discrepancy could be because of the use of a small study arena in the laboratory which may not be typical for the searching ability of the predator and the spatial complexity of an actual field [45,46,47]. Lastly, plant characteristics such as leaf hairs or trichomes can also influence the predation efficacy of a predator [48,49].

In the field, dispersal ability, prey searching potential, and the presence of other prey species can influence the predation efficacy of any predator. Due to the ability to utilize alternate food sources such as pollen, the dispersal ability of *O. insidiosus* can be influenced in the field depending on the presence or absence of flowers. A higher dispersal rate of adult *O. insidiosus* females was found in the strawberry flowers without pollen, and the least dispersal was observed in the flowers with pollen. The flowers with the main prey *F. occidentalis* result in the intermediate dispersal rate [50]. Apart from dispersal, foraging behavior is another important factor for predatory insects because it directly impacts the predator’s ability to locate prey and reduce the pest population. *Orius insidiosus* was found to be responsive to the non-volatile cue released by prey species and involved in intensive foraging behavior, with more turns in the foraging area and higher immobile time [51]. In the field, when multiple thrips species were present, *O. insidiosus* killed more *F. occidentalis* than *Frankliniella bispinosa* (Morgan) (Thysanoptera: Thripidae), because *F. bispinosa* was more mobile and smaller in size than *F. occidentalis* [52]. This can result in a change in the functional response of a predator from Type II to Type III [53].

This study helps to understand the daily predation rate and interaction of *Orius insidiosus* with *S. dorsalis* in the control environment with increased prey density. Considering that *Orius* spp. had many previous reports of successfully controlling other thrips species in open fields and in the greenhouse, this study may be the first step towards its evaluation as a potential biological control agent against *S. dorsalis* in strawberries. The range of prey density tested in this study may be expanded in future studies to test population densities above 20 *S. dorsalis* per strawberry leaf and per *O. insidiosus*. However, in the current study, this range was selected, keeping in mind the action threshold of *S. dorsalis* which typically ranges from 2 to 10 thrips per plant tissue type depending on the plant host [54,55]. This type of experiment with even higher prey densities presents a logistical challenge with handling highly mobile prey such as *S. dorsalis*.

## 5. Conclusions

Our study indicates that adult *Orius insidiosus* demonstrates a Type II functional response to both prey life stages, i.e., adult and second-instar larvae of *S. dorsalis*. In addition, adult *O. insidiosus* consumed a significantly higher number of prey than the fifth-instar nymphs in 24 h. However, further field studies are needed to understand the predator responses in the field, including foraging behavior, dispersal activity among strawberry plants, and population dynamics in the field. This study highlights the potential of *O. insidiosus* as a biological control agent for *S. dorsalis* in strawberries, offering a sustainable alternative to chemical insecticides.

## Figures and Tables

**Table 1:** Maximum likelihood estimates from logistic regression of the proportion of the second-instar larva of *S. dorsalis* eaten by adult *O. insidiosus*.

Parameter		Co-Efficient	Function Number	Estimate	STD Err	Chi-Square	Pr > Chi-Square
Intercept		P_0_ Intercept	1	−1.2203	0.1357	80.87	<0.0001
		P_0_ Intercept	2	0.1951	0.0918	4.52	0.033
Initial_prey_cat	High	P_1_ Linear	1	−0.2338	0.1601	2.13	0.1441
	High	P_1_ Linear	2	−0.2158	0.1057	4.17	0.0412
	Low	P_2_ Quadratic	1	0.6607	0.2267	8.53	0.0035
	Low	P_2_ Quadratic	2	0.7211	0.1584	20.73	<0.0001

**Table 2:** Maximum likelihood estimates from logistic regression of the proportion of the adult *S. dorsalis* eaten by adult *O. insidiosus*.

Parameter			Function Number	Estimate	STD Err	Chi-Square	Pr > ChiSq
Intercept		P_0_ Intercept	1	0.8500	0.1597	28.34	<0.0001
		P_0_ Intercept	2	0.3973	0.1701	5.46	0.0195
Initial_prey_cat	High	P_1_ Linear	1	−0.4989	0.1732	8.30	0.004
	High	P_1_ Linear	2	−2.3432	0.2238	109.60	<0.0001
	Low	P_2_ Quadratic	1	0.6698	0.2890	5.37	0.0205
	Low	P_2_ Quadratic	2	1.9053	0.2852	44.64	<0.0001

**Table 3:** Mean ± SE of attack rate (a) and handling ability (T_h_) of *O. insidiosus* on second-instar larvae and *S. dorsalis* adults.

Prey Stage	Parameters	Estimate ± SE	Asymptomatic 95% CL		R^2^
			Lower	Upper	
Second-instar larvae	a	0.0342 ± 0.0018	0.0306	0.0379	0.735
	T_h_	0.01 ± 0.00	0.011	0.01	
*S. dorsalis* adult	a	0.0335 ± 0.004	0.0248	0.0423	0.789
	T_h_	0.8135 ± 0.2419	0.3328	1.2941	

## Data Availability

Raw data from this project will be shared specifically with interested parties, if contacted via appropriate channels.

## References

[1] USDA/NASS 2023 State Agriculture Overview for Florida. https://www.nass.usda.gov/Quick_Stats/Ag_Overview/stateOverview.php?state=FLORIDA.

[2] Liburd O., Rhodes E. (2019). Management of Strawberry Insect and Mite Pests in Greenhouse and Field Crops. Strawberry—Pre- and Post-Harvest Management Techniques for Higher Fruit Quality.

[3] Panthi B., Renkema J. (2020). Managing *Scirtothrips dorsalis* Hood (Thysanoptera: Thripidae) in Florida Strawberry with Flupyradifurone. Int. J. Fruit Sci..

[4] Reddy G.P.V., Prasad V.D., Rao R.S. (1992). Relative resistance in chilli thrips *Scirtothrips dorsalis* Hood population in Andhra Pradesh to some conventional insecticides. Indian J. Plant Prot..

[5] Dale A.G., Borden M.A. (2018). Evaluation of Reduced-Risk Insecticides to Control Chilli Thrips (Thysanoptera: Thripidae) and Conserve Natural Enemies on Ornamental Plants. Fla. Entomol..

[6] Eilenberg J., Hajek A., Lomer C. (2001). Suggestions for unifying the terminology in biological control. BioControl.

[7] Isenhour D.J., Marston N.L. (1981). Seasonal cycles of *Orius insidiosus* (Hemiptera: Anthocoridae) in Missouri soybeans. J. Kans Entomol. Soc.

[8] Bosco L., Giacometto E., Tavella L. (2008). Colonization and predation of thrips (Thysanoptera: Thripidae) by *Orius* spp. (Heteroptera: Anthocoridae) in sweet pepper greenhouses in Northwest Italy. Biol. Control.

[9] Kawai A. (1995). Control of *Thrips palmi* Karny (Thysanoptera: Thripidae) by *Orius* spp. (Heteroptera: Anthocoridae) on Greenhouse Eggplant. Appl. Entomol. Zool..

[10] Funderburk J., Stavisky J., Olson S. (2000). Predation of Frankliniella occidentalis (Thysanoptera: Thripidae) in field peppers by *Orius insidiosus* (Hemiptera: Anthocoridae). Environ. Entomol..

[11] Frescata C., Mexia A. (1996). Biological Control of Thrips (Thysanoptera) by *Orius laevigatus* (Heteroptera: Anthocoridae) in Organically-Grown Strawberries. Biol. Agric. Hortic..

[12] Perera M.T.M.D.R., Senanayake N., Dissanayake D.M.I.C.B. (2021). Evaluation of *Amblyseius swirskii* (predatory mite) and *Orius leavigatus* as biological control agents of chilli thrips (Scirtothrips dorsalis). Ceylon J. Sci..

[13] Doĝramaci M., Arthurs S.P., Chen J., McKenzie C., Irrizary F., Osborne L. (2011). Management of chilli thrips *Scirtothrips dorsalis* (Thysanoptera: Thripidae) on peppers by *Amblyseius swirskii* (Acari: Phytoseiidae) and *Orius insidiosus* (Hemiptera: Anthocoridae). Biol. Control.

[14] Lattin J.D. (1999). Bionomics of the anthocoridae. Annu. Rev. Èntomol..

[15] Pehlivan S., Alinç T., Achiri T.D., Atakan E. (2020). Functional responses of two predatory bugs (Hemiptera: Anthocoridae) to changes in the abundance of *Tetranychus urticae* (Acari: Tetranychidae) and *Bemisia tabaci* (Hemiptera: Aleyrodidae). Eur. J. Entomol..

[16] Solomon M.E. (1949). The Natural Control of Animal Populations. J. Anim. Ecol..

[17] Işikber A.A. (2005). Functional response of two coccinellid predators, *Scymnus levaillanti* and *Cycloneda sanguinea*, to the cotton aphid, *Aphis gossypii*. Turk. J. Agric. For..

[18] Fathi S.A.A., Nouri-Ganbalani G. (2010). Assessing the potential for biological control of potato field pests in Ardabil, Iran: Functional responses of *Orius niger* (Wolf.) and *O. minutus* (L.) (Hemiptera: Anthocoridae). J. Pest Sci..

[19] Holling C.S. (1959). The Canadian E n t o m o l o g i s t Some Characteristics of Simple Types of Predation and Parasitism1. Can. Entomol..

[20] Fathipour Y., Maleknia B. (2016). Mite Predators. Ecofriendly Pest Management for Food Security.

[21] Alvarado P., Baltà O., Alomar O. (1997). Efficiency of four heteroptera as predators of *Aphis gossypii* and Macrosiphum euphorbiae (Hom.: Aphididae). Entomophaga.

[22] Montserrat M., Albajes R., Castane C. (2000). Functional response of four Heteropteran predators preying on greenhouse whitefly (Homoptera: Aleyrodidae) and western flower thrips (Thysanoptera: Thripidae). Environ. Entomol..

[23] Nagai K., Yano E. (2000). Predation by *Orius sauteri* (Poppius) (Heteroptera: Anthocoridae) on *Thrips palmi* Karny (Thysanoptera: Thripidae): Functional response and selective predation. Appl. Entomol. Zool..

[24] Whitaker V.M., Peres N.A., Osorio L.F., Fan Z., do Nascimento Nunes M.C., Plotto A., Sims C.A. (2019). ‘Florida Brilliance’ strawberry. HortScience.

[25] Juliano S.A. (2003). Nonlinear Curve Fitting: Predation and Functional Response Curves. Des. Anal. Ecol. Exp..

[26] Dostálková I., Kindlmann P., Dixon A.F.G. (2002). Are classical predator-prey models relevant to the real world?. J. Theor. Biol..

[27] Mouratidis A., de Lima A.P., Dicke M., Messelink G.J. (2022). Predator-prey interactions and life history of *Orius laevigatus* and *O. majusculus* feeding on flower and leaf-inhabiting thrips. Biological. Control..

[28] Isenhour D.J., Yeargan K.V. (1981). Predation by Orius insidiosus1 on the Soybean Thrips, *Sericothrips variabilis* 2: Effect of Prey Stage and Density 3. Environ. Entomol..

[29] Taghizadeh M., Irani-Nejad K.H., Iranipour S., Vahed M.M. (2018). Daily consumption and functional response of *Stethorus gilvifrons* (Coleoptera: Coccinellidae) and *Orius albidipennis* (Hemiptera: Anthocoridae) to *Tetranychus urticae* (Acari: Tetranychidae). Persian J. Acarol..

[30] Poley K., Bahlai C., Grieshop M. (2018). Functional response of generalist predators to *Halyomorpha halys* (Hemiptera: Pentatomidae) Eggs. Environ. Entomol..

[31] Hassell M.P., Lawton J.H., Beddington J.R. (1976). The Components of Arthropod Predation: I. The Prey Death-Rate. J. Anim. Ecol..

[32] Liu P., Jia W., Zheng X., Zhang L., Sangbaramou R., Tan S., Liu Y., Shi W. (2018). Predation Functional Response and Life Table Parameters of *Orius sauteri* (Hemiptera: Anthocoridae) Feeding on *Megalurothrips usitatus* (Thysanoptera: Thripidae). Fla. Entomol..

[33] Gitonga L.M., Overholt W.A., Löhr B., Magambo J.K., Mueke J.M. (2002). Functional response of *Orius albidipennis* (Hemiptera: Anthocoridae) to *Megalurothrips sjostedti* (Thysanoptera: Thripidae). Biological. Control..

[34] Pervez A., Omkar (2006). Ecology and biological control application of multicoloured Asian ladybird, *Harmonia axyridis*: A review. Biocontrol Sci. Technol..

[35] Howard D.F., Blum M.S., Fales H.M. (1983). Defense in thrips: Forbidding fruitiness of a lactone. Science.

[36] Alves-Silva E., Del-Claro K. (2014). Fire triggers the activity of extrafloral nectaries, but ants fail to protect the plant against herbivores in a neotropical savanna. Arthropod Plant Interact..

[37] O’Neil R.J. (1989). Comparison of Laboratory and Field Measurements of the Functional Response of *Podisus maculiventris* (Heteroptera: Pentatomidae). J. Kans. Entomol. Soc..

[38] Wiedenmann R.N., O’Neil R.J. (1991). Searching behavior and time budgets of the predator *Podisus maculiventris*. Entomol. Exp. Appl..

[39] Mohaghegh, Clercq D., Tirry (2001). Functional response of the predators *Podisus maculiventris* (Say) and *Podisus nigrispinus* (Dallas) (Het., Pentatomidae) to the beet armyworm, *Spodoptera exigua* (Hübner) (Lep., Noctuidae): Effect of temperature. J. Appl. Entomol..

[40] van Lenteren J.C. (2012). The state of commercial augmentative biological control: Plenty of natural enemies, but a frustrating lack of uptake. BioControl.

[41] Pervez A., Omkar (2005). Functional responses of coccinellid predators: An illustration of a logistic approach. J. Insect Sci..

[42] Bielza P., Balanza V., Cifuentes D., Mendoza J.E. (2020). Challenges facing arthropod biological control: Identifying traits for genetic improvement of predators in protected crops. Pest Manag. Sci..

[43] Lee J.H., Kang T.J. (2004). Functional response of *Harmonia axyridis* (Pallas) (Coleoptera: Coccinellidae) to *Aphis gossypii* Glover (Homoptera: Aphididae) in the Laboratory. Biol. Control.

[44] O’Neil R.J. (1997). Functional Response and Search Strategy of *Podisus maculiventris* (Heteroptera: Pentatomidae) Attacking Colorado Potato Beetle (Coleoptera: Chrysomelidae). Environ. Èntomol..

[45] Murdoch W.W. (1973). The functional response of predators. J. Appl. Ecol..

[46] Kareiva P., Mackauer M., Ehler L.E., Roland J. (1990). The spatial dimension in pest–enemy interaction. Critical Issues in Biological Control.

[47] Uiterwaal S.F., DeLong J.P. (2018). Multiple factors, including arena size, shape the functional responses of ladybird beetles. J. Appl. Ecol..

[48] Skirvin D.J., Fenlon J.S. (2001). Plant species modifies the functional response of *Phytoseiulus persimilis* (Acari: Phytoseiidae) to *Tetranychus urticae* (Acari: Tetranychidae): Implications for biological control. Bull. Entomol. Res..

[49] Riddick E.W., Simmons A.M. (2014). Do plant trichomes cause more harm than good to predatory insects?. Pest Manag. Sci..

[50] Alonso M., Guisoni N., Rocca M., Greco N. (2024). To move or not to move: Dispersal of *Orius insidiosus* in strawberry plants. Entomol. Exp. Appl..

[51] Traczyk E., Funderburk J., Martini X. (2020). Foraging behavior responses of *Orius insidiosus* to thrips cues. Entomol. Exp. Appl..

[52] Reitz S.R., Funderburk J.E., Waring S.M. (2006). Differential predation by the generalist predator *Orius insidiosus* on congeneric species of thrips that vary in size and behavior. Entomol. Exp. Appl..

[53] Lehtinen S.O., Perälä T.A., Uusi-Heikkilä S.K., Kuparinen A.K. (2024). Mutually exclusive feeding yields Holling type III functional response. Funct. Ecol..

[54] Nault B.A., Shelton A.M. (2010). Impact of insecticide efficacy on developing action thresholds for pest management: A case study of onion thrips (Thysanoptera: Thripidae) on onion. J. Econ. Entomol..

[55] Gill H.K., Garg H., Gill A.K., Gillett-Kaufman J.L., Nault B.A. (2015). Onion thrips (Thysanoptera: Thripidae) biology, ecology, and management in onion production systems. J. Integr. Pest Manag..

